# GPR88 in D1R-Type and D2R-Type Medium Spiny Neurons Differentially Regulates Affective and Motor Behavior

**DOI:** 10.1523/ENEURO.0035-19.2019

**Published:** 2019-08-08

**Authors:** A. C. Meirsman, Sami Ben Hamida, E. Clarke, A. de Kerchove d’Exaerde, E. Darcq, B. L. Kieffer

**Affiliations:** 1 Département de Médecine Translationnelle et Neurogénétique, Institut de Génétique et de Biologie Moléculaire et Cellulaire, Institut National de la Santé et de la Recherche Médicale Unité 964, Centre National de la Recherche Scientifique Unité Mixte de Recherche 7104, Université de Strasbourg, Illkirch, France; 2 Neuroscience Paris Seine, Institut de Biologie Paris Seine, Centre National de la Recherche Scientifique Unité Mixte de Recherche 8246/Institut National de la Santé et de la Recherche Médicale Unité 1130/Université Pierre et Marie Currie, Paris F-75005, France; 3 Douglas Research Center, Department of Psychiatry, McGill University, Montréal, Canada; 4 Laboratory of Neurophysiology, Université Libre de Bruxelles (ULB), ULB Neuroscience Institute, 1070 Brussels, Belgium

**Keywords:** anxiety, locomotion, medium spiny neuron, motor coordination, orphan GPCR, striatum

## Abstract

The orphan receptor GPR88 is highly expressed in D1 receptor (D1R)- and D2R-medium spiny neurons (MSNs) and has been associated to striatum-dependent functions in rodents. The total deletion of *Gpr88* in mice was shown to decrease anxiety-like behaviors, increase stereotypies and locomotion, and impair motor coordination and motor learning. Knowing the opposing role of D1R- and D2R-MSNs, we here investigated the respective roles of GPR88 in the two MSN subtypes for these behaviors. To do so, we compared effects of a conditional *Gpr88* gene knock-out (KO) in D1R-MSNs (D1R-*Gpr88* mice) or D2R-MSNs (A_2A_R-*Gpr88* mice) with effects of the total *Gpr88* KO (CMV-*Gpr88* mice). Overall, most phenotypes of CMV-*Gpr88* mice were recapitulated in A_2A_R-*Gpr88* mice, including reduced marble burying, increased social interactions, increased locomotor activity and stereotypies in the open field, and reduced motor coordination in the rotarod. Exceptions were the reduced habituation to the open field and reduced motor skill learning, which were observed in CMV-*Gpr88* and D1R-*Gpr88* mice, but not in A_2A_R-*Gpr88* mice. D1R-*Gpr88* mice otherwise showed no other phenotype in this study. Our data together show that GPR88 modulates the function of both D1R- and D2R-MSNs, and that GPR88 activity in these two neuron populations has very different and dissociable impacts on behavior. We suggest that GPR88 in D2R-MSNs shapes defensive and social behavior and contributes in maintaining the inhibition of basal ganglia outputs to control locomotion, stereotypies and motor coordination, while GPR88 in D1R-MSNs promotes novelty habituation and motor learning.

## Visual Abstract

**Figure:**
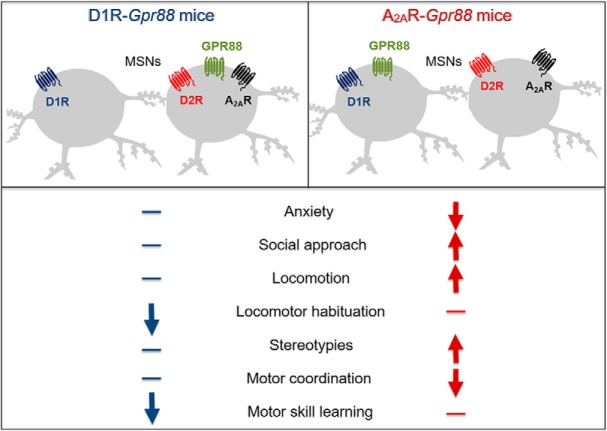

## Significance Statement

GPR88, an orphan G-protein-coupled receptor (GPCR), has been implicated in the regulation of striatum-dependent behaviors. In the striatum, GPR88 is most abundant in both medium spiny neurons (MSNs)-expressing dopamine D1 and D2 receptors. We compared effects of a conditional *Gpr88* gene knock-out (KO) in D1 receptor (D1R)-MSNs or D2R-MSNs with effects of the total *Gpr88* deletion. Our data suggest that GPR88 in D2R-MSNs shapes defensive and social behavior and contributes in maintaining the inhibition of basal ganglia outputs to control locomotion, stereotypies and motor coordination, while GPR88 in D1R-MSNs promotes novelty habituation and motor learning. *Gpr88* therefore plays very distinct roles in modulating D1R-type and D2R-type neurons function and the related behaviors.

## Introduction

Among brain orphan G-protein-coupled receptors (GPCRs), GPR88 shows highest and almost restricted expression in the striatum, a key region in motor control, cognitive functions and motivational processes (Liljeholm and O'Doherty, 2012; Quintana et al., 2012; Ehrlich et al., 2018). Homozygous deleterious mutation of *Gpr88* in humans was linked to a familial developmental disorder characterized by a childhood chorea (hyperkinetic movement disorder), learning disabilities and marked speech retardation (Alkufri et al., 2016). Previous reports have shown that mice lacking *Gpr88* present hyperlocomotion, increased stereotypies, motor coordination and motor learning deficits (Logue et al., 2009; Quintana et al., 2012; Meirsman et al., 2016b). The total *Gpr88* gene deletion in mice also induced failure to habituate to an open field or automated home-cage environment and decreased anxiety-like behaviors (Meirsman et al., 2016b; Maroteaux et al., 2018). Additionally, AAV-mediated re-expression of GPR88 in the dorsal striatum [caudate putamen (CPu)] restored the locomotor hyperactivity and motor learning deficits in knock-out (KO) animals, thus providing a direct link between GPR88 loss in the dorsal striatum and the locomotor phenotype of KO mice (Quintana et al., 2012; Meirsman et al., 2016b).

Within the striatum, GPR88 is expressed in the majority of medium spiny neurons (MSNs) of both the direct [co-expressing dopamine D1 receptors (D1Rs) and substance P, D1R-MSNs] and indirect (co-expressing dopamine D2Rs, adenosine A_2A_ receptor (A_2A_R) and Enkephalin, D2R-MSNs] pathways (Quintana et al., 2012). Converging evidence support the opposing influence of these two MSNs populations in motor output systems and motivated behavior. For example, optogenetic depolarization of D2R-MSNs decreased locomotor initiation (Kravitz et al., 2010), while ablation or disruption of these neurons increased motor activity (Durieux et al., 2009, 2012; Bateup et al., 2010). In contrast, optical stimulation of D1R-MSNs increased locomotion whereas disruption or ablation of these neurons had the opposite effect (Kravitz et al., 2010; Durieux et al., 2012). Also, cell-specific neuron ablation using an inducible diphtheria toxin receptor (DTR)-mediated cell targeting strategy further suggests a differential role of D2R- and D1R-MSNs in acquisition and expression of motor skill learning (Durieux et al., 2012). Ablation of D2R-MSNs neurons delayed the acquisition of a rotarod task but had no effect in a previously acquired motor skill. Contrarily, ablation of D1R-MSNs neurons impaired motor skill learning regardless of the training extension and also disrupted performance of a previously learned motor sequence (Durieux et al., 2012). Further, in recent years, research on MSNs subtypes function has revealed that these two neuronal populations differentially regulate not only motor behaviors but also responses to rewarding and aversive stimuli: while optogenetic activation of the D1R-MSNs was shown to increase reinforcement, activation of D2R-MSNs induced transient punishment and depressive-like behavior (Kravitz et al., 2012; Hikida et al., 2013; Francis et al., 2015).

Despite the established overall function of striatal GPR88 in brain functions and deficits (in humans and mice), no study to date has directly compared the specific role of GPR88 in D1R-and D2R-MSNs. A conditional KO mouse line for GPR88 in D2R-MSNs was developed in a previous study, using a A_2A_R-Cre driver line (A_2A_R*-Gpr88* mice), and mutant mice showed hyperactive behavior, decreased anxiety-like behaviors and increased locomotor response to dopaminergic agonists (Meirsman et al., 2016a, 2017). In this study, we have generated conditional *Gpr88* KO for D1R-MSNs (D1R-*Gpr88* mice), and compared behavioral responses of D1R-*Gpr88* with those of A_2A_R*-Gpr88* mice and total KO (CMV-*Gpr88*) mice. Results show that GPR88 in D1R neurons regulates locomotor habituation to novel environments and motor skill learning. In contrast, GPR88 in D2R, but not in D1R neurons, control defensive burying and social approach, and also regulate levels of locomotion, stereotypies and initial motor coordination.

## Materials and Methods

### Subjects

Mice (male and female) aged 9–15 weeks where bred in house and grouped-house three to five animals per cage. Animals where maintained on a 12/12 h light/dark cycle at controlled temperature (22 ± 1°C). Food and water were available ad libitum throughout all experiments.

### Generation of mutant mice

*Gpr88*-floxed mice, total *Gpr88* KO (CMV-*Gpr88*) and A_2A_R*-Gpr88* mice were produced as previously described (Meirsman et al., 2016a,b). To generate CMV-*Gpr88*, *Gpr88*-floxed mice (C57BL/6 background) were crossed with CMV-Cre mice (50%-C57BL/6J; 50%-129/sv) expressing Cre recombinase under the cytomegalovirus promoter. To generate a conditional KO of *Gpr88* in D2R-MSNs (A_2A_R*-Gpr88*) or D1R-MSNs (D1R*-Gpr88*) *Adora2a*-Cre (Durieux et al., 2009) and *Drd1a*-Cre (gensat.org; congenic on C57BL/6J) mice were, respectively, crossed with *Gpr88*-floxed mice (Meirsman et al., 2016b). First generation animals expressing the Cre under the control of A_2A_R or D1R promotor (*Gpr88^A2AR-Cre/+^* and *Gpr88 ^D1R-Cre/+^*) were crossed a second time to eliminate the wild-type *Gpr88* gene. We therefore generated 3 mouse lines with different mixed genetic background.

For all experiments, and considering the different genetic background, A_2A_R*-Gpr88* and D1R*-Gpr88* mice were compared to their *Gpr88*-floxed littermates (A_2A_R-CTL and D1R-CTL, respectively) and CMV-*Gpr88* mice were compared to their wild-type controls (CMV-CTL). Baseline responses may therefore slightly differ when comparing the three mouse colonies.

### Tissue preparation and fluorescent *in situ* hybridization

RNAscope was used as previously described (Meirsman et al., 2016a). Mice (*n* = 3 D1R-CTL; *n* = 3 D1R*-Gpr88*) were killed by cervical dislocation and fresh brains were extracted and embedded in optimal cutting temperature (OCT) medium (Thermo Scientific) frozen and kept at –80°C. Frozen brains were coronally sliced into 20-µm serial sections by using cryostat (CM3050 Leica), placed in superfrost slides (Thermo Scientific) and kept at –80°C until processing. *In situ* hybridizations were performed using the RNAscope Multiplex Fluorescent Assay. GPR88 and D1R probes were alternatively coupled to FITC or TRITC while D2R probes were coupled with Cy5.

### Relative expression of D1R and D2R mRNA in GPR88-positive cells

Image acquisition was performed using the slide scanner Olympus VS120 (Olympus Corporation). Regions of interest (ROIs) were selected using Olyvia software (Olympus) and saved as PNG files. Three brain regions where analyzed: rostral CPu (from 1.42 to 0.98 mm from bregma), caudal CPu (from 0.98 to –0.58 mm from bregma), and nucleus accumbens (Nacc; from 1.42 to 1.10 mm from bregma).

For the CPu (rostral and caudal), at least four ROIs were selected: two for the dorso-lateral striatum (DLS) and two for dorso-median striatum (DMS). Counting was balanced between right and left hemispheres. To evaluate expression of D1R and D2R mRNA in GPR88-expressing cells, counting was performed manually using the FIJI (ImageJ) cell counter. First, cells expressing GPR88 mRNA were marked and counted (±55 cells/ROI in D1R-CTL mice; ±21 cells/ROI in D1R-*Gpr88* mice). For each GPR88-positive cell, co-expression of D1R or D2R was verified and counted separately. Relative co-expression (GPR88/D1R or GPR88/D2R) is represented as a percentage of total GPR88-positive cells counted [(number of GPR88-expressing cells co-expressing D1R or D2R × 100)/total number of GPR88-expressing cells]. Statistical analysis where realized with percentages of each ROI calculated using excel. Given the lack of difference in GPR88 expression between lateral and medial CPu, relative percentage of each was pooled for graphical representation and statistical analysis.

### [S35]-GTPγS binding assay

[S35]-GTPγS assays were performed on membrane preparations as described in previous report (Pradhan et al., 2009). To perform [S35]-GTPγS assays on whole striatum mice were killed by cervical dislocation and both striatum were rapidly manually removed, frozen in dry ice, and stored at –80°C until use. Two (CMV-*Gpr88* and CMV-CTL) and three (D1R-*Gpr88* and D1R-CTL) membrane preparations were used. Each membrane preparation was generated using striatum from three animals (males and females). Results are expressed by meaning measures from the three-membrane preparation. All assays were performed on membrane preparations. Membranes were prepared by homogenizing the tissue in ice-cold 0.25 M sucrose solution 10 vol (ml/g wet weight of tissue). Samples were then centrifuged at 2500 × *g* for 10 min. Supernatants were collected and diluted 10 times in buffer containing 50 mM TrisHCl (pH 7.4), 3 mM MgCl2, 100 mM NaCl, and 0.2 mM EGTA, following which they were centrifuged at 23,000 × *g* for 30 min. The pellets were homogenized in 800-μl ice-cold sucrose solution (0.32 M) and kept at –80°C. For each [35S]GTPγS binding assay 2 μg of protein per well was used. Samples were incubated with and without ligands, for 1 h at 25°C in assay buffer containing 30 mM GDP and 0.1 nM [35S]GTPγS. Bound radioactivity was quantified using a liquid scintillation counter. *B*_max_ and *K*_d_ values were calculated. Non-specific binding was defined as binding in the presence of 10 μM GTPγS and binding in the absence of agonist was defined as the basal binding.

### Gene expression analysis

Mice were killed by cervical dislocation. Brains structures (Nacc *n* = 8 D1R-CTL; *n* = 7 D1R-*Gpr88* and CPu *n* = 9 D1R-CTL; *n* = 9 D1R-*Gpr88,* hippocampus: *n* = 9 D1R-CTL; *n* = 7 D1R-*Gpr88* and amygdala: *n* = 6 D1R-CTL; *n* = 7 D1R-*Gpr88*) from D1R-*Gpr88* and controls were quickly dissected out, frozen on dry ice and stored at −80°C until used. RNA was isolated using TRIzol reagent (Invitrogen) following the manufacturer’s instructions. cDNA was synthetized using the first-strand Superscript II kit (Invitrogen, Life Technologies). Quantitative real-time PCR (qRT-PCR) was performed in triplicates on a LightCycler 480 RT- PCR (Roche) and SyberGreen masterMix (Roche). Thermal cycling parameters were 1 min at 95°C followed by 40 amplification cycles of 15 s at 95°C, 15 s at 60°C, and 30 s at 72°C. Relative expression ratios were normalized to the level of actin and the 2−ΔΔCt method was applied to assess differential expression level of GPR88.

### Behavioral experiments

For all behavioral measures, mice from different mouse lines and genotypes were tested in a random order and data were analyzed blind to genotype. However, to avoid order-related variability between mouse lines and genotypes, mouse lines were stratified so that each session included both genotypes from all lines. One mouse cohort (*N* = 8 CMV-CTL, *N* = 10 CMV-*Gpr88; N* = 10 A_2A_R*-Gpr88*, *N* = 10 A_2A_R*-*CTL; *N* = 10 D1R*-Gpr88*, *N* = 14 D1R*-*CTL) was used to measure marble burying and social interaction. Independent cohorts of mice (*N* = 21 CMV-CTL, *N* = 21 CMV-*Gpr88*; *N* = 10 A_2A_R*-Gpr88*, *N* = 17 A_2A_R*-*CTL; *N* = 13 D1R*-Gpr88*, *N* = 12 D1R*-*CTL) underwent 5 d of open field locomotion followed by a 48-h resting period before 7 d of rotarod motor skill learning.

### Marble burying

Defensive burying was measured as previously described (Meirsman et al., 2016b) using the marble burying test conducted with 20 small glass marbles (15 mm) evenly spaced in a transparent single cage (21 × 11 × 17 cm) over 4-cm sawdust bedding. The cage was covered by a plastic lid in a room illuminated at 40 Lux. Mice were left in the cage for 10 min, and the number of marbles buried more than half in sawdust was counted.

### Social interaction test

Social interaction was assessed, as previously described (Meirsman et al., 2016a), in an open field (50 × 50 cm) dimly lit (<10 Lux) using naive wild-type mice of the same age and weight as interactors. On the first day, all mice were individually placed in the open field arena and left for a 10-min period of habituation. The next day, mice were placed in the open field arena with a wild-type naive interactor and a 10-min session was recorder. Nose contacts were measured manually using video recordings. If an interactor failed to engage in any interaction data from the respective mice were exclude from analysis.

### Open field locomotion

To assess basal locomotion and habituation to novel environment mice were placed in a dimly lit open field (Accuscan Instruments) for 30-min daily session. The experiment lasted 5 d, and mice were placed in the same open field for all sessions tested. Open field was cleaned with water and 70% ethanol between trials. Total distance traveled, stereotypy counts, and durations were automatically recorded.

### Rotarod

The first day, mice were placed on the rod (30-mm plastic roller, Panlab Harvard) at constant speed of 4 rpm. until achieving 90 s without falling from the rod (habituation; data not shown). On the six next consecutive days, mice were placed on the rod accelerating from 4 to 40 rpm in 5 min and the remaining at maximum speed for the next 5 min for four trials every day. Light intensity in the room was inferior to 10 Lux. Mice rested a minimum of 1 min between trials to avoid fatigue and exhaustion. When mice hang on the rod instead of running, they were left for one complete turn but the timer was stopped if the mice engaged in a second consecutive turn. Animals were scored for their latency to fall (in seconds) in each trial. Mean values of four daily trials were used for statistical analysis.

### Statistics

For *in situ* hybridization cell counting and GPR88 agonist-induced binding assay data were analyzed using two-way ANOVA followed by Sidak’s and Tukey’s multiple comparisons, respectively. Repeated measures (RM) two-way ANOVA was used to analyze global open field and rotarod results with genotypes as the between-subject factor and time as the RM. One-way ANOVA was used for open field habituation analysis (days 1 and 5), first and last rotarod session analysis. Method of contrasts was used to compare day 1 and day 6 performance on the rotarod. Stereotypies, marble burying and social interaction contacts were analyzed using *t* test (unpaired with Welch’s correction). All statistical analyses were realized using GraphPad Prism 7 (GraphPad Software, Inc) and the accepted level of significance was *p* < 0.05. All the statistical methods are summarized in Table 1.

**Table 1.** Detailed statistical analysis

				ANOVA			*t* test
Assay	Mouse line	Number	Figure	Genotype effect	Cell type/time/treatment	Interaction	
RT-qPCR	D1R-*Gpr88*	*N* = 9 D1R-CTL; *N* = 9 D1R-*Gpr88*	1*A* (CPu)				*t*_(16)_ = 3.01, *p* = 0.008
		*N* = 8 D1R-CTL; *N* = 7 D1R-*Gpr88*	1*A* (Nacc)				*t*_(13)_ = 4.19, *p* = 0.001
		*N* = 9 D1R-CTL; *N* = 7 D1R-*Gpr88*	1*A* (Hipp)				*t*_(14)_ = 2.7, *p* = 0.017
		*N* = 6 D1R-CTL; *N* = 7 D1R-*Gpr88*	1*A* (Amy)				*t*_(11)_ = 0.53, *p* = 0. 6
[35S]-GTPgS binding	CMV-*Gpr88*; D1R-*Gpr88*	*N* = 3 D1R-CTL, D1R-*Gpr88*, CMV-CTL, CMV-*Gpr88*	1*B*	*F*_(3,66)_ = 185.2; *p* < 0.0001	*F*_(10,66)_ = 95.64; *p* < 0.0001	*F*_(30,66)_ = 23.19; *p* < 0.0001	
*In situ* hybridization/cell counting	D1R-*Gpr88*	*N* = 3 D1R-CTL, *N* = 3 D1R-*Gpr88*	2*B* (CPu)	*F*_(1,134)_ = 0.4164; *p* = 0.5198	*F*_(1,134)_ = 957.2; *p* < 0.0001	*F*_(1,134)_ = 387.8; *p* < 0.0001	
			2*B* (Nacc)	*F*_(1,36)_ = 0.2597; *p* = 0.6134	*F*_(1,36)_ = 204.3; *p* < 0.0001	*F*_(1,36)_ = 97.83; *p* < 0.0001	
Marble burying	CMV-*Gpr88*	*N* = 9 CMV-CTL, *N* = 10 CMV-*Gpr88*	3*A*, left				*t*_(17)_ = 2.03, *p* = 0.059
	D1R-*Gpr88*	*N* = 14 D1R-CTL, *N* = 10 D1R-*Gpr88*	3*B*, left				*t*_(22)_ = 1.002, *p* = 0.33
	A2AR-*Gpr88*	*N* = 10 A2AR-CTL, *N* = 10 A2AR-*Gpr88*	3*C*, left				*t*_(18)_ = 4.01, *p* < 0.001
Nose contact in social interaction	CMV-*Gpr88*	*N* = 8 CMV-CTL, *N* = 8 CMV-*Gpr88*	3*D*, right				*t*_(14)_ = 2.88, *p* = 0.012
	D1R-*Gpr88*	*N* = 14 D1R-CTL, *N* = 8 D1R-*Gpr88*	3*E*, right				*t*_(20)_ = 2.57, *p* = 0.018
	A2AR-*Gpr88*	*N* = 10 A2AR-CTL, *N* = 10 A2AR-*Gpr88*	3*F*, right				*t*_(18)_ = 2.06, *p* = 0.01
Open field (all sessions)	CMV-*Gpr88*	*N* = 21 CMV-CTL, *N* = 21 CMV-*Gpr88*	4*A*, left	*F*_(1,40)_ = 4.357; *p* = 0.0425	*F*_(4,180)_ = 4.419; *p* = 0.002	*F*_(4,180)_ = 7.189; *p* < 0.0001	
	D1R-*Gpr88*	*N* = 13 D1R-CTL, *N* = 12 D1R-*Gpr88*	4*B*, left	*F*_(1,23)_ = 1.106; *p* = 0.3038	*F*_(4,92)_ = 31.03; *p* < 0.0001	*F*_(4,92)_ = 11.82; *p* < 0.0001	
	A2AR-*Gpr88*	*N* = 17 A2AR-CTL, *N* = 10 A2AR-*Gpr88*	4*C*, left	*F*_(1,25)_ = 8.004; *p* = 0.0091	*F*_(4,100)_ = 43.28; *p* < 0.0001	*F*_(4,100)_ = 3.939; *p* = 0.0052	
Open field (sessions 1 and 5)	CMV-*Gpr88*	*N* = 21 CMV-CTL, *N* = 21 CMV-*Gpr88*	4*A*, right	*F*_(1,80)_ = 4.93; *p* = 0.0292	*F*_(1,80)_ = 1.25;*p* = 0.2679	*F*_(1,80)_ = 8.94; *p* = 0.0037	
	D1R-*Gpr88*	*N* = 13 D1R-CTL, *N* = 12 D1R-*Gpr88*	4*B*, right	*F*_(1,46)_ = 0.78; *p* = 0.3811	*F*_(1,46)_ = 26.75;*p* < 0.0001	*F*_(1,46)_ = 11.01; *p* = 0.0018	
	A2AR-*Gpr88*	*N* = 17 A2AR-CTL, *N* = 10 A2AR-*Gpr88*	4*C*, right	*F*_(1,50)_ = 8.17; *p* = 0.0062	*F*_(1,50)_ = 18.71; *p* < 0.0001	*F*_(1,50)_ = 0.1479; *p* = 0.7021	
Stereotypies	CMV-*Gpr88*	*N* = 21 CMV-CTL, *N* = 21 CMV-*Gpr88*	5*A*				Score: *t*_(40)_ = 2.228; *p* = 0.0316Time: *t*_(40)_ = 2.818; *p* = 0.0075
	D1R-*Gpr88*	*N* = 13 D1R-CTL, *N* = 12 D1R-*Gpr88*	5*B*				Score: *t*_(23)_ = 1.156; *p* = 0.2594Time: *t*_(23)_ = 0.7174; *p* = 0.4803
	A2AR-*Gpr88*	*N* = 17 A2AR-CTL, *N* = 10 A2AR-*Gpr88*	5*C*				Score: *t*_(25)_ = 2.291; *p* = 0.0307Time: *t*_(25)_ = 2.317; *p* = 0.0290
Rotarod (all sessions)	CMV-*Gpr88*	*N* = 21 CMV-CTL, *N* = 21 CMV-*Gpr88*	6*A*, left	*F*_(1,40)_ = 17.73; *p* = 0.0001	*F*_(23,920)_ = 13.49; *p* < 0.0001	*F*_(23,920)_ = 3.159; *p* < 0.0001	
	D1R-*Gpr88*	*N* = 13 D1R-CTL, *N* = 12 D1R-*Gpr88*	6*B*, left	*F*_(1,23)_ = 8.759; *p* = 0.0070	*F*_(23,529)_ = 10.09; *p* < 0.0001	*F*_(23,529)_ = 7.607; *p* < 0.0001	
	A2AR-*Gpr88*	*N* = 17 A2AR-CTL, *N* = 10 A2AR-*Gpr88*	6*C*, left	*F*_(1,25)_ = 8.008; *p* = 0.0091	*F*_(23,575)_ = 13.74; *p* < 0.0001	*F*_(23,575)_ = 1.017; *p* = 0.4403	
Rotarod (sessions 1 and 6)	CMV-*Gpr88*	*N* = 21 CMV-CTL, *N* = 21 CMV-*Gpr88*	6*A*, right	*F*_(1,80)_ = 32.62; *p* < 0.0001	*F*_(1,80)_ = 17.67 *p* < 0.0001	*F*_(1,80)_ = 4.517; *p* = 0.0367	
	D1R-*Gpr88*	*N* = 13 D1R-CTL, *N* = 12 D1R-*Gpr88*	6*B*, right	*F*_(1,46)_ = 13.62; *p* = 0.0006	*F*_(1,46)_ = 11.15; *p* = 0.0017	*F*_(1,46)_ = 7.643; *p* = 0.0082	
	A2AR-*Gpr88*	*N* = 17 A2AR-CTL, *N* = 10 A2AR-*Gpr88*	6*C*, right	*F*_(1,50)_ = 8.067; *p* = 0.0065	*F*_(1,50)_ = 16.31; *p* = 0.0002	*F*_(1,50)_ = 1.299; *p* = 0.2598	

## Results

### *D1R-Gpr88* mice show *Gpr88* mRNA deletion in D1R-expressing neurons

**Figure 1.**
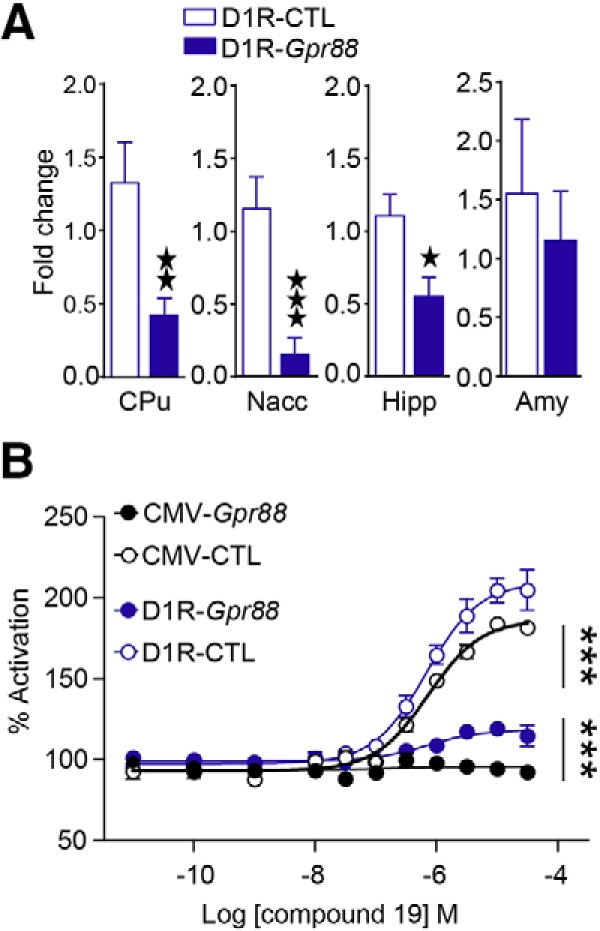
GPR88 agonist-induced activation and mRNA levels in D1R-*Gpr88* mice. We measured levels of *Gpr88* mRNA in D1R-CTL and D1R-*Gpr88* mice (***A***) and show a significant reduction of GPR88 expression in the CPu, Nacc, hippocampus (Hipp), and amygdala (Amy). We also performed GPR88-mediated [35S]-GTPγS assay (***B***) and show that protein activation was totally and partially abolished in the striatum of CMV-*Gpr88* and D1R-*Gpr88* mice, respectively. Two (CMV-*Gpr88* and control mice) and three (D1R-*Gpr88* and control mice) membrane preparations were used per genotype. Data are presented as mean ± SEM. ***A***, CPu: *n* = 9 D1R-CTL; *n* = 9 D1R-*Gpr88*; Nacc: *n* = 8 D1R-CTL; *n* = 7 D1R-*Gpr88*; Hipp: *n* = 9 D1R-CTL; *n* = 7 D1R-*Gpr88;* Amy: *n* = 6 D1R-CTL; *n* = 7 D1R-*Gpr88*; two black stars *p* < 0.01; three black stars *p* < 0.001 (Welch’s *t* test). ***B***, *n* = 3 D1R-CTL; *n* = 3 D1R-*Gpr88*; *n* = 2 CMV-Gpr88 and *n* = 2 CMV-CTL. Three text stars *p* < 0.001 Tukey’s multiple comparisons of D1R-CTL or CMV-CTL versus D1R-*Gpr88* and CMV-*Gpr88* versus D1R-*Gpr88*.

To conditionally delete *Gpr88* exon 2 in cells expressing D1R, mice carrying two LoxP sites flanking the second exon of the *Gpr88* gene (Meirsman et al., 2016b) were crossed with mice expressing the Cre recombinase under the control of the *Drd1a* gene promoter (Gensat). We first tested whether GPR88 transcript and protein are reduced in the striatum. We quantified *Gpr88* mRNA levels by RT-qPCR for CPu and Nacc from D1R-*Gpr88* and their control littermates. As shown in Figure 1*A*, *Gpr88* expression was significantly decreased in striatal regions of conditional KO compared to controls (CPu: *t*_(16)_ = 3.01, *p* = 0.008; Nacc: *t*_(13)_ = 4.19, *p* = 0.001; Table 1). Testing *Gpr88* mRNA levels in the hippocampus and amygdala showed a milder but significant reduction in hippocampus but not amygdala (Hipp: *t*_(14)_ = 2.7, *p* = 0.017; Amy *t*_(11)_ = 0.53, *p* = 0.6), indicating that GPR88 KO may also have occurred in some extrastriatal regions containing D1R-type neurons (see Discussion). There was no significant difference in D1R expression levels across genotypes (data not shown). To establish whether reduced mRNA level translates into lower protein level, we tested GPR88 signaling in the striatum. To this aim, we performed GPR88 agonist-induced [S35]-GTPγS binding assays (Fig. 1*B*) with membranes prepared from whole striatum (CPu and Nacc) of D1R-*Gpr88* mice and their controls, as well as with total KO CMV-*Gpr88* mice (negative control) and their wild-type control mice (positive control). Two-way RM ANOVA revealed a significant genotype effect (*F*_(3,66)_ = 185.2, *p* < 0.0001) and interaction effect (*F*_(30,66)_ = 23.19, *p* < 0.0001). *Post hoc* analysis (Tukey’s multiple comparisons) revealed significant differences (*p* < 0.0001) between D1R-*Gpr88* mice (118.4 ± 1.17%) and their control littermates (209.5 ± 2.47%) as well as between D1R-*Gpr88* mice and CMV-*Gpr88* mice (95.61 ± 1.72%; *p* < 0.0001). This result confirms that the *Drd1a*-Cre-driven conditional *Gpr88* gene deletion produced a significant reduction of GPR88 expression. Importantly, deletion of GPR88 does not affect the function of D1R. Indeed, locomotor response to D1R agonist SKF 81297 is comparable in D1R-*Gpr88* mice and their corresponding controls (data not shown).

**Figure 2.**
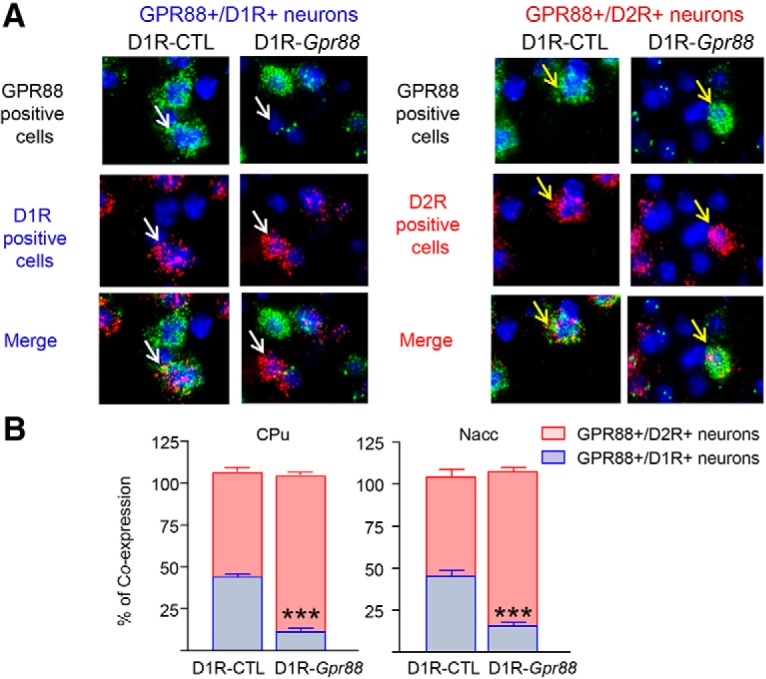
Molecular characterization of conditional D1R-*Gpr88* mice. *Gpr88*, *Drd1a* and *Drd2* mRNA expression in the CPu of D1R-CTL (left panels) and D1R-*Gpr88* (right panels) mice using triple fluorescent *in situ* hybridization (***A***). *Gpr88* is labeled in green (FITC), *Drd1a* (left panels) in red (TRITC) and *Drd2* (right panels) in red (Cy5). In D1R-CTL animals, *Gpr88* mRNA colocalizes with both *Drd2* and *Drd1a* mRNA. In contrast, *Drd2* but not *Drd1a* colocalize with *Gpr88* mRNA in D1R-*Gpr88* mice. White and yellow arrows indicate examples of *Drd1a*-positive and *Drd2*-positive cells, respectively. DAPI staining (blue) was used to label all cells nuclei. Quantification of *Gpr88/Drd2* (red) and *Gpr88/Drd1a* (blue) mRNA co-expression in the CPu and Nacc (***B***) of D1R-*Gpr88* and control mice (*n* = 3/genotype). Colocalization of *Gpr88* and *Drd1a* mRNA was significantly decreased in the CPu and Nacc of D1R-*Gpr88* mice compared to control littermates (Sidak’s multiple comparison; *p* < 0.0001). Percentage of co-expression was calculated based on the total number of *Gpr88*-positive cells counted [(number *Gpr88*-expressing cells co-expressing *Drd1a* or *Drd2* × 100)/total number of *Gpr88*-expressing cells]. Data are presented as mean ± SEM. ***B***, *n* = 3 D1R-CTL; *n* = 3 D1R-*Gpr88*. Text stars: three stars *p* < 0.001 (Sidak’s multiple comparison of *Gpr88/Drd1a* co-expression between genotypes).

We then tested whether the genetic deletion was specific to D1R MSNs, using *in situ* hybridization. As depicted in Figure 2*A*, we first demonstrate that, in control mice cells expressing *Gpr88* mRNA colocalize with both *Drd1a* (left panel), and *Drd2* mRNA-expressing cells (right panel). In D1R-*Gpr88* mice, however, cells expressing *Gpr88* do not colocalize with *Drd1a-*expressing cells (left panel), but still colocalize with *Drd2-*expressing cells (right panel). Quantitative analysis (Fig. 2*B*) in the CPu and Nacc confirmed that, in control animals, *Gpr88* mRNA is found in both *Drd1a-*positive (CPu: 43.96 ± 1.54% and Nacc: 45.13 ± 3.57%) and *Drd2-*positive (CPu: 62.11 ± 1.95% and Nacc: 59.03 ± 4.47%) cells whereas in D1R-*Gpr88* mice the great majority of cells expressing *Gpr88* are found in *Drd2-*expressing cells (CPu: 92.83 ± 1.45% and Nacc: 91.88 ± 2.48%) with significantly reduced number of cells co-expressing *Drd1a* mRNA (CPu: 11.16 ± 1.43% and Nacc: 15.50 ± 2.18%; CPu; genotype: *F*_(1,134)_ = 0.42; *p* = 0.52; cell type: *F*_(1,134)_ = 957.2; *p* < 0.0001; interaction: *F*_(1,134)_ = 387.8, *p* < 0.0001; Nacc; genotype: *F*_(1,36)_ = 0,26, *p* = 0,6134; cell type: *F*_(1,36)_ = 204,3, *p* < 0,0001; interaction: *F*_(1,36)_ = 97.83, *p* < 0.0001; *n* = 3/genotype), indicating that the *Gpr88* deletion had occurred mostly in D1R-type MSNs.

### A_2A_R*-Gpr88* but not D1R-*Gpr88* mice show altered defensive burying and social approach

**Figure 3.**
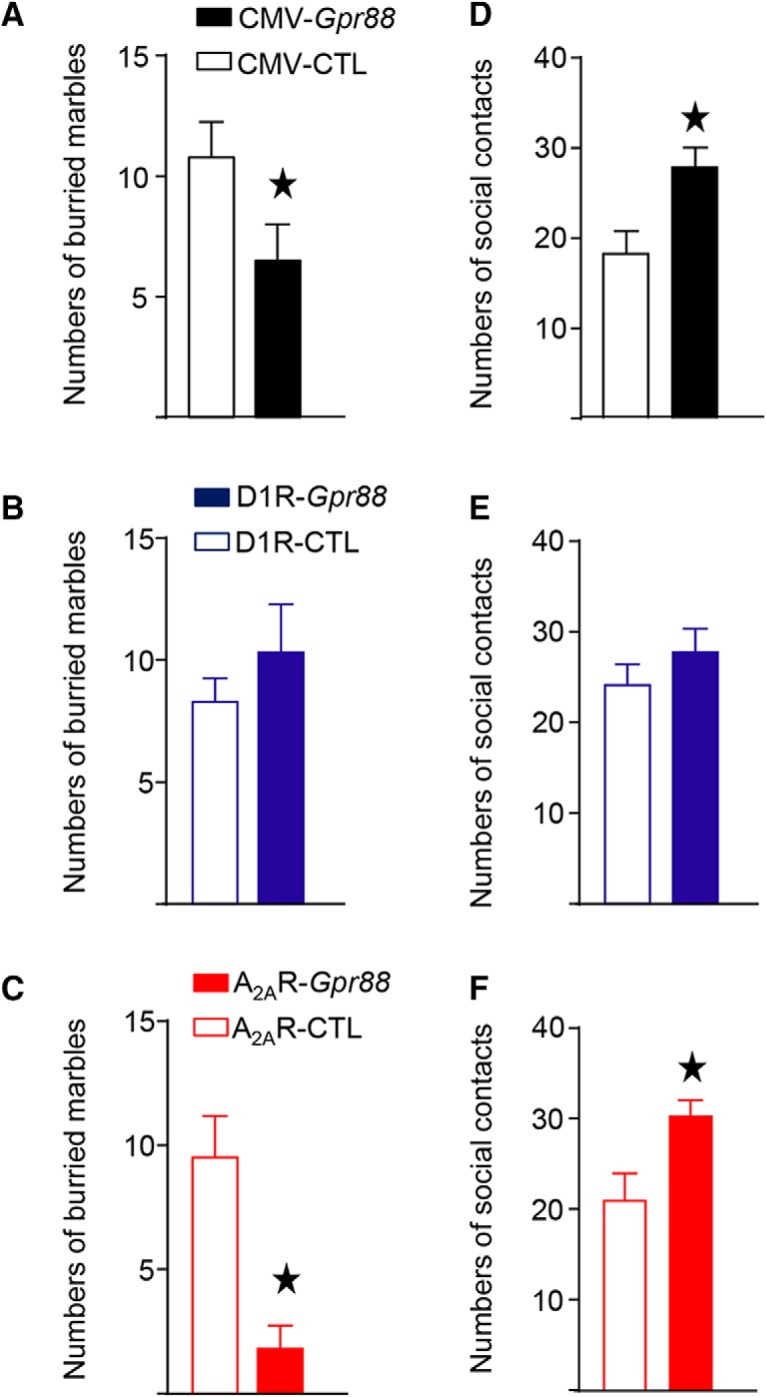
CMV-*Gpr88* and A_2A_R*-Gpr88* but not D1R-*Gpr88* mice show altered defensive burying and social behavior. When placed in the presence of 20 marbles CMV-*Gpr88* (***A***) and A_2A_R-*Gpr88* (***C***) mice buried less marbles than control animals. D1R-*Gpr88* mice (***B***) show similar numbers of buried marbles compared to control animals. To test social behaviors all mice where left in the presence of a naive wild-type congener and nose contact was counted. Once again, both CMV-*Gpr88* (***D***) and A_2A_R-*Gpr88* (***F***) mice but not D1R-*Gpr88* mice (***E***) showed increased number of nose contacts compared to their littermates. Data are represented as mean ± SEM. ***A***, ***D***, *n* = 8 CMV-CTL, *n* = 10 CMV-*Gpr88*. ***B***, ***E***, *N* = 14 D1R-CTL, *N* = 10 D1R-*Gpr88*. ***C***, ***F***, *n* = 10 A_2A_R-CTL; *n* = 10 A_2A_R-*Gpr88*. Black stars: one star *p* < 0.05 (Welch’s *t* test).

The deletion of *Gpr88* specifically in D2R-neurons is sufficient to decrease anxiety-like behaviors and increase social approach (Meirsman et al., 2016a). Here, we investigated whether deletion of *Gpr88* in D1R-neurons also modifies anxiety-related and/or social behaviors. As depicted in Figure 3*A–C*, using the defensive burying paradigm we first confirmed that CMV-*Gpr88* (*t*_(17)_ = 2.03, *p* = 0.059; *n* = 9–10) buried less marbles than their control littermates. D1R-*Gpr88* mice showed equal numbers of buried marbles compared to D1R-CTL mice (*t*_(22)_ = 1.002, *p* = 0.33; *n* = 10–14), whereas A_2A_R-*Gpr88* mice, like CMV-*Gpr88*, showed reduced number of buried marbles (*t*_(18)_ = 4.01, *p* < 0.001; *n* = 10/genotype). Also, in the presence of a naive, wild-type, congener (Fig. 3*D–F*), CMV-*Gpr88* (*t*_(14)_ = 2.88, *p* = 0.012; *n* = 8/genotype), and A_2A_R-*Gpr88* mice (*t*_(18)_ = 2.06, *p* = 0.01; *n* = 10/genotype) showed increased numbers of nose contacts but D1R-*Gpr88* mice displayed similar numbers of contacts than their control littermates (*t*_(20)_ = 2.57, *p* = 0.018; *n* = 10–14).

The present results confirm previous findings (Meirsman et al., 2016a) that *Gpr88* deletion in D2R-neurons is sufficient to recapitulate emotional and social phenotypes observed in CMV-*Gpr88* mice and reveal that deletion of *Gpr88* in D1R-neurons does not alter neither defensive marble burying or social approach.

### A_2A_R-*Gpr88* mice show hyperlocomotion, whereas D1R-*Gpr88* mice show lack of habituation in a novel environment

**Figure 4.**
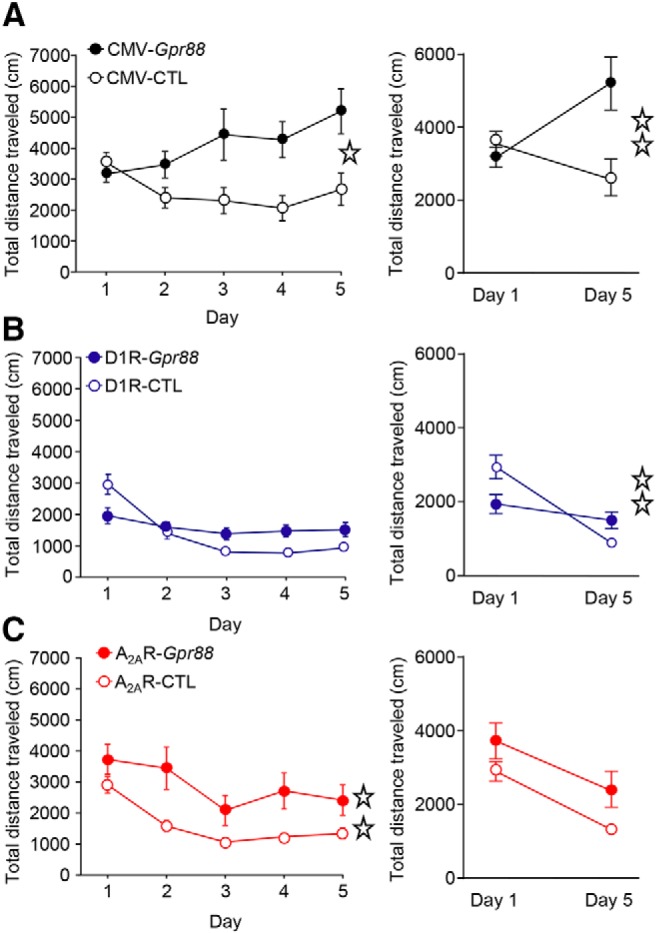
Locomotor activity is increased in A2AR-*Gpr88* mice whereas D1R-*Gpr88* mice show lack of locomotor habituation. When placed individually in a dimly lit open field for 30-min daily sessions during 5 d, both CMV-*Gpr88* (***A***) and A_2A_R-*Gpr88* (***C***) but not D1R-*Gpr88* (***B***) mice traveled a longer distance then their control littermates. D1R-*Gpr88* mice, however, present similar total locomotion when compared to their control littermates (***B***). When comparing locomotion between the first (1) and last session (5), CMV-*Gpr88* mice, in contrast to CMV-CTL, traveled a longer distance in the last compared to the first day. In contrast to their control littermates, D1R-*Gpr88* mice show similar locomotion in the first and last open field session. Regardless of their hyperlocomotion, A_2A_R-*Gpr88* mice habituated to the open field presenting decreased overall locomotion in the last test session. Line graphs show the distance traveled (cm) in 5-min bins over a 30-min session. Bar graphs show the average total distance traveled (cm) over the 30-min sessions period. Data are represented as mean ± SEM. ***A***, *n* = 21 CMV-CTL; *n* = 21 CMV-*Gpr88*. ***B***, *N* = 13 D1R-CTL, *N* = 12 D1R-*Gpr88*. ***C***, *n* = 17 A_2A_R-CTL; *n* = 10 A_2A_R-*Gpr88*. Open stars: one star *p* < 0.05; two stars *p* < 0.01 (RM two-way ANOVA).

Previous studies showed that mice lacking *Gpr88* display increased spontaneous locomotor activity as well as lack of habituation to a novel environment (Quintana et al., 2012; Meirsman et al., 2016b; Maroteaux et al., 2018). Deletion of *Gpr88* in D2R expressing neurons was further shown sufficient to recapitulate the hyperlocomotion phenotype observed in CMV-*Gpr88* mice (Meirsman et al., 2016a). Here, we tested whether GPR88 in D1R and D2R MSNs play a differential role in the regulation of locomotor and exploratory behavior. To do this, CMV-*Gpr88*, A_2A_R-*Gpr88*, and D1R-*Gpr88* mice and their corresponding controls were individually placed in a dimly lit open field chambers during five successive daily 30-min sessions. Analysis of total locomotion confirmed a significantly increased locomotor activity for CMV-*Gpr88* mice (two-way RM ANOVA; genotype: *F*_(1,40)_ = 5.98, *p* = 0.0189; day: *F*_(4,180)_ = 4.42; *p* = 0.002; interaction: *F*_(4,180)_ = 7.19; *p* < 0.0001; *n* = 21/genotype; Fig. 4*A*, left panel). Further, while control animals decreased their general locomotion between the first and last session (see Fig. 4*A*, right panel), CMV-*Gpr88* mice showed rather increased locomotion in the last compared to the first session (two-way ANOVA; genotype: *F*_(1,80)_ = 4.93, *p* = 0.029; day: *F*_(1,80)_ = 1.25, *p* = 0.27; interaction: *F*_(1,80)_ = 8.94, *p* = 0.0037). D1R-*Gpr88* mice (Fig. 4*B*, left panel) presented similar levels of general locomotor activity when compared to their littermates (two-way RM ANOVA; genotype: *F*_(1,23)_ = 1.11, *p* = 0.30; day: *F*_(4,92)_ = 31.03; *p* < 0.0001; interaction: *F*_(4,92)_ = 11.82; *p* < 0.0001; *n* = 12–13) but, similar to CMV-*Gpr88* mice, showed lack of locomotor habituation to the open field environment (two-way ANOVA; genotype: *F*_(1,46)_ = 0.78, *p* = 0.38; day: *F*_(1,46)_ = 26.75, *p* < 0.0001; interaction: *F*_(1,46)_ = 11.01, *p* = 0.0018; Fig. 4*B*, right panel). Similar to CMV-*Gpr88*, A_2A_R-*Gpr88* mice (Fig. 4*C*, left panel) significantly increased their locomotion when compared to control littermates (two-way RM ANOVA; genotype: *F*_(1,25)_ = 8.0, *p* = 0.009; day: *F*_(4,100)_ = 43.28; *p* < 0.0001; interaction: *F*_(4,100)_ = 3.94; *p* = 0.005; *n* = 10–17). These mice, however, showed equal locomotor habituation profile than their littermates with decreased locomotion in the last compared to the first open field session (right panel; two-way ANOVA; genotype: *F*_(1,50)_ = 8.17, *p* = 0.006; day: *F*_(1,50)_ = 18.71, *p* < 0.0001; interaction: *F*_(1,50)_ = 0.15, *p* = 0.70).

These results first confirm that deletion of *Gpr88* increases general locomotion and simultaneously abolishes locomotor habituation to a novel environment. Further, our results suggest that deletion of D1R-*Gpr88* does not impact general locomotion but abolishes locomotor habituation to a novel environment. In contrast, deletion of *Gpr88* in D2R-MSNs increases locomotor activity without altering habituation to a novel environment.

### A_2A_R-*Gpr88* but not D1R-*Gpr88* mice show increased stereotypies in the open field

**Figure 5.**
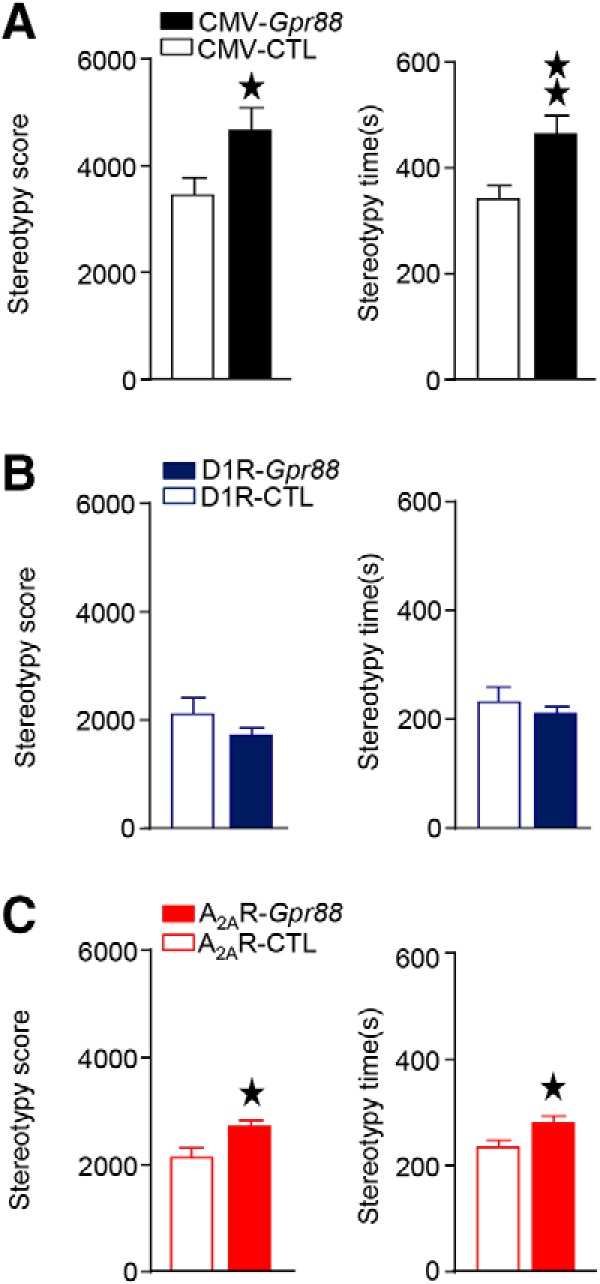
CMV-*Gpr88* and A_2A_R-*Gpr88* gene deletion increases stereotypies. When placed in an open field for 30 min (day 1), CMV-*Gpr88* (***A***) and A_2A_R-*Gpr88* (***C***) present increased number and duration of stereotypies. D1R-*Gpr88* mice (***B***), however, show no difference in the number or time spent in stereotypies when compared to their control littermates. Data are represented as mean ± SEM. ***A***, *n* = 21 CMV-CTL; *n* = 21 CMV-*Gpr88*. ***B***, *N* = 13 D1R-CTL, *N* = 12 D1R-*Gpr88*. ***C***, *n* = 17 A_2A_R-CTL; *n* = 10 A_2A_R-*Gpr88*. black stars: one star *p* < 0.05; two stars *p* < 0.01 (Student’s *t* test).

Previous studies indicate increased repetitive motor behaviors or stereotypies (Logue et al., 2009; Meirsman et al., 2016b), as well as increased perseverative behavior (Maroteaux et al., 2018) in CMV-*Gpr88* mice. To examine whether this phenotype results from *Gpr88* deletion in D1R- and/or D2R-MSNs, we analyzed stereotypies scores in the first open field session (30 min). Results indicate that both CMV-*Gpr88* (Fig. 5*A*) and A_2A_R-*Gpr88* mice (Fig. 5*C*) presented higher stereotypies score (*t*_(40)_ = 2.23; *p* = 0.031; *n* = 21/genotype and *t*_(25)_ = 2.29; *p* = 0.031; *n* = 10–17, respectively) and increased stereotypy time (*t*_(40)_ = 2.82; *p* = 0.007; and *t*_(25)_ = 2.32; *p* = 0.029, respectively). On the contrary, D1R-*Gpr88* mice (Fig. 5*B*) presented no altered stereotyped behavior when compared to control animals.

These results show that GPR88 in D2R-expressing but not D1R-expressing neurons regulates motor stereotypies.

### A_2A_R-*Gpr88* mice show impaired motor coordination, whereas D1R-*Gpr88* mice show lack of motor skill learning

**Figure 6.**
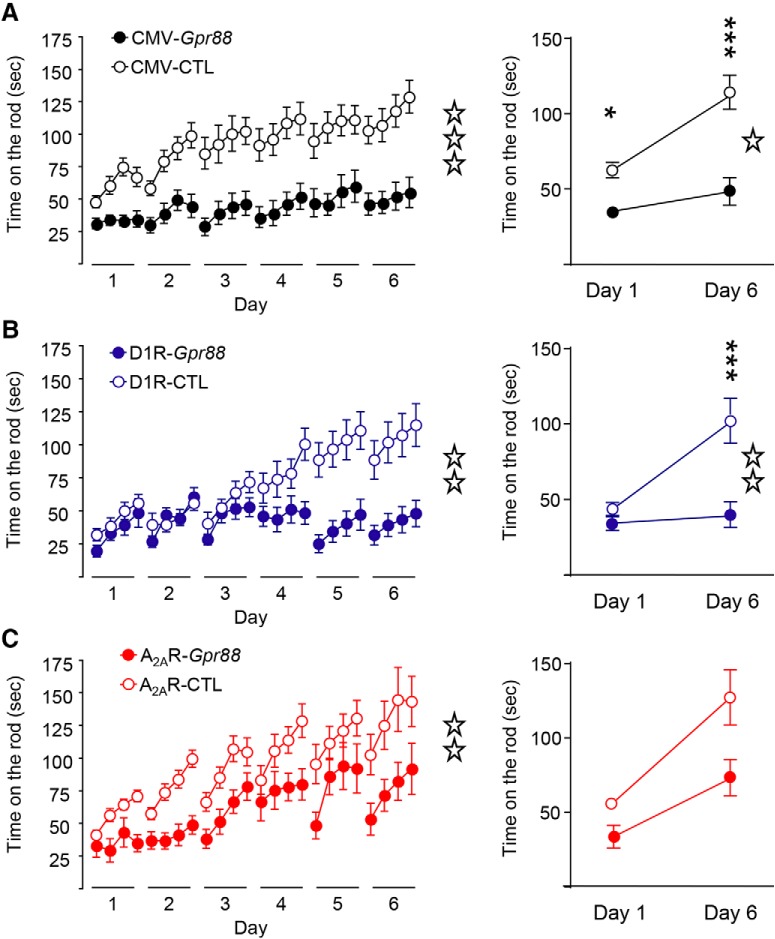
Motor coordination deficits in A_2A_R-*Gpr88* mice and motor skill learning deficits in D1R-Gpr88 KO mice. Mice where tested on a rotating rod for four daily trials lasting 6 d. Overall (left panel), CMV-*Gpr88* (***A***), D1R-*Gpr88* (***B***), and A_2A_R-*Gpr88* (***C***) mice show decreased latency to fall form the rod. When selectively analyzing the first and last training session (right panel), we observe that CMV-*Gpr88* and A_2A_R-*Gpr88* mice presented motor coordination deficits as soon as the first session which is not present in D1R-*Gpr88*. In the last session, however, CMV-*Gpr88* and D1R-*Gpr88* both present a significantly decreased time on the rod when compared to control mice, whereas A_2A_R-*Gpr88* mice present no significant difference in the time spent on the rod when compared to their littermates. Data are represented as mean ± SEM. ***A***, *n* = 21 CMV-CTL; *n* = 21 CMV-*Gpr88*. ***B***, *N* = 13 D1R-CTL, *N* = 12 D1R-*Gpr88*. ***C***, *n* = 17 A_2A_R-CTL; *n* = 10 A_2A_R-*Gpr88*. Open stars: one star *p* < 0.05; two stars *p* < 0.01; three stars *p* < 0.001 (RM two-way ANOVA). Text stars: three stars *p* < 0.001; one star *p* < 0.05 (Sidak’s multiple comparison).

CMV-*Gpr88* mice have been previously shown to present initial motor coordination deficits coupled with abolished motor skill learning throughout the rotarod tasks (Quintana et al., 2012; Meirsman et al., 2016b). We therefore compared motor coordination and motor skill learning performances of CMV-, D1R-and A_2A_R-*Gpr88* mice by testing them in a rotating rod for six consecutive daily sessions. As depicted in Figure 6*A*, left panel, two-way RM ANOVA confirmed an impaired motor coordination for CMV-*Gpr88* mice (genotype: *F*_(1,40)_ = 17.73, *p* < 0.0001; day: *F*_(23,920)_ = 13.49, *p* < 0.0001; *n* = 21/genotype) as well as a significant genotype × time effect (*F*_(23,920)_ = 3.16; *p* < 0.0001) confirming the lack of motor skill learning as previously published (Quintana et al., 2012; Meirsman et al., 2016b). In fact, CMV-*Gpr88* mice show decreased motor coordination in day 1 and maintained poor performance until the end of the task (day 6; right panel; two-way ANOVA; genotype: *F*_(1,80)_ = 32.62, *p* < 0.0001, day: *F*_(1,80)_ = 17.67, *p* < 0.0001; interaction: *F*_(1,80)_ = 4.52, *p* = 0.0367). *Post hoc* analysis revealed significant differences between CMV-Gpr88 mice and control animals during day 1 (*p* < 0.05) and 6 (*p* < 0.0001). In addition, only control animals showed an improved motor performance between days 1 and 6 (*p* < 0.0001). Similarly, D1R-*Gpr88* mice (Fig. 6*B*, left panel) presented significantly decreased motor learning performance (two-way RM ANOVA; genotype: *F*_(1,23)_ = 8.76, *p* = 0.007; day: *F*_(23,529)_ = 10.09, *p* < 0.0001; *n* = 12–13) and significant genotype × time effect (*F*_(23,529)_ = 7.61; *p* < 0.0001). Despite similar motor coordination than their control littermates on day 1, D1R-*Gpr88* mice failed to learn the task presenting decreased time on the rod on day 6 compared to their littermates (right panel; two-way ANOVA; genotype: *F*_(1,46)_ = 13.62, *p* = 0.0006; day: *F*_(1,46)_ = 11.15, *p* = 0.0017; interaction: *F*_(1,46)_ = 7.64, *p* = 0.008). *Post hoc* analysis revealed significant differences between D1R-*Gpr88* mice and control animals only during day 6 (*p* < 0.0001). Moreover, only control animals showed an improved motor performance between days 1 and 6 (*p* < 0.001). A_2A_R-*Gpr88* mice (Fig. 6*C*, left panel) on the other hand also presented significantly decreased global performance (two-way RM ANOVA; genotype: *F*_(1,25)_ = 8.01, *p* = 0.0091; day: *F*_(23,575)_ = 13.74; *p* < 0.0001; interaction: *F*_(23,575)_ = 1.02; *p* = 0.44; *n* = 10–17) but show motor learning skills over days of experiment. In fact, when comparing day 1 and day 6 (right panel), two-way ANOVA showed significant genotype (*F*_(1,50)_ = 8.07, *p* = 0.0065) and day (*F*_(1,50)_ = 16.31, *p* = 0.0002) effect but not interaction: *F*_(1,50)_ = 1.29, *p* = 0.2598). Subsequent analyses using the method of contrasts showed that both genotypes improved their performance in the accelerating rotarod between day 1 and day 6 (A_2A_R-CTL: *p* = 0.001; A_2A_R-*Gpr88 p* = 0.018).

These data first confirm that lack of GPR88 abolishes both motor coordination and motor skill learning and further shows that the deletion of A_2A_R-*Gpr88* alters initial motor coordination while preserving motor skill learning, whereas D1R-*Gpr88* deletion selectively impairs motor skill learning.

### Discussion

Results from the comparison of total versus conditional mouse lines are summarized in Table 2. In sum, data from marble burying and social interaction tests reveal a D2R cell-specific function of GPR88 in anxiety-related and social behavior (De Boer and Koolhaas, 2003), as modifications are detected in CMV-*Gpr88* and A_2A_R-*Gpr88* KO, but not D1R-*Gpr88* KO, mice. With regards to open field results, we observe differential roles of GPR88 in D1R- and D2R-MSNs, suggesting that GPR88 in D1R-MSNs has no role on general locomotion or stereotypies but regulates locomotor habituation to a novel environment, whereas deletion of this receptor in D2R-MSNs increases spontaneous locomotion and stereotypies while preserving locomotor habituation. In the rotarod also, we show differential roles of GPR88 in D1R- and D2R-MSNs, indicating that GPR88 in D1R-MSNs contributes to motor skill learning, whereas the receptor in D2R-MSNs contributes to motor coordination but not learning in the task. Overall therefore, our study demonstrates that GPR88 modulates the function of both D1R- and D2R-MSNs and that GPR88 activity in these two neuron populations has very different and dissociable impacts on behavior.

**Table 2.** Summary of behavioral phenotypes observed in CMV-*Gpr88*, D1R-*Gpr88*, and A_2A_R-*Gpr88* mice

		**CMV-*Gpr88***	**D1R-*Gpr88***	**A_2A_R-*Gpr88***
Marble burying		↓	↔	↓
Social interaction		↑	↔	↑
Open field	Locomotion	↑	↔	↑
	Habituation	↓	↓	↔
	Stereotypies	↑	↔	↑
Rotarod	Motor coordination	↓	↔	↓
	Motor skill learning	↓	↓	↔

We find that specific deletion of *Gpr88* in D2R-MSNs, but not in D1R-MSNs, decreases anxiety-like behavior as shown by reduced defensive burying activity (Borsini et al., 2002; De Boer and Koolhaas, 2003; Meirsman et al., 2016a). This result is in line with data showing that blocking D2R-MSNs activity disrupts avoidance behavior and aversive learning (Hikida et al., 2013). We also extend previous data (Meirsman et al., 2016a) by showing that *Gpr88* deletion in D2R but not D1R-neurons increases social approach. Reports have shown that dopamine signaling through D1Rs is necessary for mediating pro-social behavior (Gunaydin et al., 2014). Also, it was shown that while D1R-MSNs display reduced mEPSC frequency after chronic social defeat, optical stimulation of D1R-MSNs was sufficient to reverse social avoidance induced by social defeat stress (Francis et al., 2015; Francis and Lobo, 2017). Therefore, although baseline social approach is not affected by D1R-*Gpr88* deletion, different results may be obtained after chronic social stress. Knowing that inducible ablation of D1R-MSNs also reduces anxiety behaviors in mice (Révy et al., 2014), our result suggest that D1R-MSNs and D1R‘s-dependent anxiety and social behaviors was not affected by D1R-*Gpr88* ablation. However, to fully understand GPR88 function in affective and social behaviors future studies should compare responses of D1R-*Gpr88* and A_2A_R -*Gpr88* mice in reward and aversive learning paradigms and investigate the role of this orphan receptor in stress-induced social avoidance.

We then show a differential effect of *Gpr88* gene KO in D1R- or D2R-MSNs on general locomotion, with hyperlocomotor activity observed after D2R-*Gpr88* deletion only. Converging data show that disruption of D2R-MSNs activity results in hyperlocomotor behavior (Durieux et al., 2012; Révy et al., 2014) while ablation of D1R-MSNs decreases locomotion (Durieux et al., 2012; Révy et al., 2014). Therefore, the increased locomotion observed in CMV-*Gpr88* and A_2A_R-*Gpr88* mice could simply result from decreased D2R-MSNs driven inhibition of locomotor output. Although deletion of *Gpr88* in D1R-neurons did not alter overall locomotion throughout the five sessions, D1R-*Gpr88* mice displayed decreased acute locomotor activity during the first open field session which would suggests impaired D1R-MSNs activity. Overall, locomotion results suggest that lack A_2A_R-*Gpr88* mimics D2R-MSNs ablation (Durieux et al., 2009, 2012; Bateup et al., 2010; Révy et al., 2014). The question of how *Gpr88* cell-specific deletion affects MSNs firing activity and basal ganglia output remains open, and future electrophysiological studies should measure basal ganglia output in D2R-*Gpr88* and D1R-*Gpr88* mice.

Another interesting locomotor phenotype in the open field is the lack of intersession habituation to the environment selectively observed in D1R-*Gpr88* mice. Open field habituation is described as an adaptive process in which rodents decrease their locomotion with increasing exposure to the same environment and is taken as an index of memory (Tomaz et al., 1990; Cerbone and Sadile, 1994). A previous study showed that total deletion of *Gpr88* improved spatial learning and memory tasks performances, thus suggesting that the non-habituating phenotype is not linked to spatial memory functions (Meirsman et al., 2016b). Surprisingly, our results contrast with the lack of open field habituation previously observed after ablation of D2R-MSNs (but not D1R-MSNs; Durieux et al., 2012). Therefore, in opposite to locomotion results, deletion of GPR88 in D1R-MSNs matches results obtained after D2R-MSNs ablation, suggesting either MSNs cross talk or alteration of a common network shaping locomotor habituation. In fact, data show (Sanguedo et al., 2016) that locomotor habituation to novel environments is accompanied by activation of striatal and extra-striatal regions such as amygdala and frontal cortex. Accordingly, CMV-*Gpr88* mice have been shown to have altered transcriptional profiles in these structures where both GPR88 and D1R are expressed (Meirsman et al., 2016b). Most importantly, recent studies using CMV-*Gpr88* mice have shown impaired multisensory processing (Ehrlich et al., 2018) and sensorimotor gating (Meirsman et al., 2017) that, coupled with altered sensorimotor and cortico-striatal functional connectivity (Arefin et al., 2017), suggest a role of this receptor in the integration and processing of sensory information. Interestingly, it has also been suggested that modifications of the striato-cortical circuitry may underlie the hyperactivity observed in CMV*-Gpr88* mice (Arefin et al., 2017). As such, future studies measuring functional connectivity in D2R-*Gpr88* and D1R-*Gpr88* mice will elucidate how cell-specific deletion *of Gpr88* reshape brain connectome leading to persistent changes in behavior.

Finally, the open field observations also reveal that A_2A_R-*Gpr88* but not D1R-*Gpr88* mice present increased number of stereotypies in the open field. Animal and clinical data indicate that dysregulation of cortico-striato-thalamo-cortical circuitry are associated with stereotypies (Lewis and Kim, 2009). Further, one study linked decreased D2R-MSNs activity with enhances stereotypies (Tanimura et al., 2010, 2011) and a recent report indicates that increasing D2R-MSNs activity is sufficient to rescue repetitive behaviors observed in a genetic model of autism (Wang et al., 2017). The increased stereotypies of A_2A_R-*Gpr88* mice may therefore result from diminished D2R-MSNs inhibitory projection. As for locomotion result, the electrophysiological impact of *Gpr88* specific deletion should be assessed in future studies. On the other hand, stereotypies have been linked to dopaminergic overstimulation (Katherine, 2018), which could also cause the phenotype observed in A_2A_R-*Gpr88* mice. In fact, we have previously reported altered DA levels in the CPu and midbrain nuclei of CMV-*Gpr88* mice, and future studies should verify DA levels in conditional *Gpr88* KO mice.

As for the open field experiments, rotarod testing also reveals differential D1R- versus D2R-MSNs activities of GPR88. Mutants lacking *Gpr88* in D1R-neurons present similar initial rotarod performance than control animals but show absence of motor skill learning throughout 6 d of task. On the contrary, mice lacking *Gpr88* in D2R-neurons show decreased latency to fall in the first day but learned the task and increased their motor performances across days. Interestingly, as for the locomotor phenotype, results are comparable to those obtained after inducible ablation of D1R-MSNs and D2R-MSNs (Durieux et al., 2012). Worth noting, previous reports indicate that *Gpr88* deletion does not alter striatal cell population or cytoarchitectural organization (Logue et al., 2009; Quintana et al., 2012) but increased levels of striatal pDARPP-32 Thr-34 and the ratio of pDARPP-32 Thr-34/DARPP-32 suggesting compromised MSNs functioning (Logue et al., 2009). Also, mRNA levels of genes encoding neurotransmitter receptors as well as GPCRs activation were found altered in the striatum of CMV-*Gpr88* mice (Quintana et al., 2012; Meirsman et al., 2016b). In particular, *Gpr88* deletion increased mu opioid and delta opioid receptors activation in the striatum. These receptors are known to activate Gi/o pathways, and could therefore contribute to increase MSNs hyperpolarization in *Gpr88* mutant mice (Le Merrer et al., 2013; Pellissier et al., 2018). Interestingly, a previous report showed that chronic administration of DOR antagonist (naltrindole) in CMV-*Gpr88* mice reversed their initial motor coordination impairment suggesting that increased DOR activity may underlie the deficit observed in A_2A_R-*Gpr88.* Future studies pharmacologically tackling receptors known to interact with GPR88 will elucidate MSNs-specific GPR88 interactions with other receptors.

In conclusion, the present study demonstrates dissociable roles of GPR88 at the level of MSNs. While GPR88 in D2R-MSNs regulates levels of anxiety, social behavior, stereotypies, locomotion, and motor coordination, this receptor in D1R-MSNs does not seem to impact affective behaviors but regulates habituation to novelty and motor skill learning. It is important to note that in the present study deletion of *Gpr88* is not exclusively striatal. Thus, a new approach to restrict D1R-Gpr88 deletion to the striatum will determine if extra-striatal structures are involved in the phenotypes observed in mutants lacking *Gpr88* in D1R-neurons. In addition, cellular mechanisms underlying phenotypes observed in this study remain to be clarified. Interesting to note, behavioral analyses show that both the total ablation of D2R MSNs (Durieux et al., 2012) and the deletion of *Gpr88* in D2R-neurons (our study) reduce motor coordination and induces hyperlocomotion, suggesting that GPR88 activity normally stimulates D2R-MSNs. This is counterintuitive, as GPR88 has been proposed to be an inhibitory GPCR (Jin et al., 2018). Also, Quintana et al. (2012) have previously shown that total GPR88 ablation reduced tonic GABA current and enhanced glutamatergic signaling in MSNs. They also showed that deletion of *Gpr88* similarly affect the response to cortical excitatory input or the tonic GABA currents in D1R or D2R MSNs. We may, however, consider a strong differential effect of selective versus total deletion of GPR88 on MSNs intrinsic electrical properties. Therefore, electrophysiological studies using cell-specific *Gpr88* deletion and also the precise anatomic localization of the receptor at presynaptic or postsynaptic levels should help clarifying how GPR88 modulates D1R- and D2R-MSNs activities. In addition, deficient long distance communication between brain structures observed in CMV-*Gpr88* mice (Arefin et al., 2017) may explain some of the present results and upcoming studies should compare respective functional connectivity alterations in the two conditional *Gpr88* KO mouse lines. Hence, further dissection of D1R versus D2R specific GPR88 activities is essential to explore the full potential of this receptor as a target for affective and motor disorders.

## Synthesis

Reviewing Editor: Carmen Sandi, Swiss Federal Institute of Technology Decisions are customarily a result of the Reviewing Editor and the peer reviewers coming together and discussing their recommendations until a consensus is reached. When revisions are invited, a fact-based synthesis statement explaining their decision and outlining what is needed to prepare a revision will be listed below. The following reviewer(s) agreed to reveal their identity: Deveroux Ferguson.

The two reviewers and editor consider that this manuscript reports a well-designed and performed study, with sound results and discussion. In previous studies, the orphan receptor GPR88 had been described to regulate striatal medium spiny neuron activity and to participate in several striatum-dependent and -independent behaviors but its contribution in a cell type-specific manner in dopamine receptor D1R- or D2R- MSNs was elusive. Here, using Gpr88KO and D1- or D2-Gpr88KO mice, the authors provide novel data on the role of Gpr88 in dopamine receptor specific cells. Overall, the manuscript is well written. This informative and comprehensive characterization of the behavioral contribution of specific subsets of Gpr88-expressing cells, pave the way for future studies on underlying molecular mechanisms. We only have a few minor points to raise to the authors:

- The authors emphasize the striatal nature of their results. However, although GPR88 is highly enriched in the striatum, its expression is also abundant in other areas (such as cortex, some subcortical nuclei and brainstem). Similarly, Drd1a is also expressed in cortex, brainstem and other nuclei (i.e hypothalamus). Hence, to convincingly demonstrate a striatal origin of the behavioral alterations described, authors should show either that GPR88 expression is not affected in other regions (i.e. cortex/brainstem) in D1R-Gpr88 mice or, alternatively, to perform an approach to restrict D1R-Gpr88 deletion to the striatum. In case the authors have good reasons not to add these analyses, we urge them to include a clear limitation for the interpretation of their data in the Discussion section.

- In light of their results, the authors suggest that GPR88 ablation in D2R-expressing MSN equates to D2-MSN inactivation, pointing at a stimulatory role for GPR88 in D2R-expressing MSNs. The authors discuss that this would be in apparent conflict with the suggested inhibitory role for GPR88 as described by Jin et al (2018) and that detailed electrophysiological studies are required to assess D1R- and D2R-MSN function in the context of GPR88 deficiency. While this conclusion is sound, the authors should take into consideration that it has been shown how global GPR88 deficiency leads to reduced GABAergic signaling and enhanced glutamatergic signaling in MSNs, leading to overall increased MSN activity (Quintana et al., 2012). Therefore, we urge the authors to acknowledge this point in the Discussion section, even though these studies did not study individual D1R- and D2R- responses, which may be underlying the observed (potential) discrepancies.

- The authors conclude that only GPR88 in D2-MSNs are related to effects on social behavior. Actually, both MSN subtypes have distinct roles in social avoidance behaviors in the chronic social defeat model (Francis and Lobo, 2017, Biol Psychiatry 81:645). We urge the authors to explain/discuss the social behavior results deeper.

Figures:

- In the Figure 1, signal intensity and number of D1R positive cells of D1R-Gpr88 (Fig.1F) are relatively lower than control groups. It is recommended that the authors use better representative images for the D1R-Gpr88.

- Figure 1B seems to be mislabeled (CMV-CTL should respond while CMV-Gpr88 should show no response).

- In Figure 2, GPR88 positive cells in D1R-Gpr88 mice seem to have higher intensity than in CTL mice. Do the authors know if there is a compensatory increase in Gpr88 expression in non-D1R Grp88-expressing cells? Along the same lines, do authors have data on behavioral response to repeated D1R activation (i.e. sensitization)?

- In Fig. 5 (as in several other figures for other parameters), the baselines of stereotypic behavior are clearly different between CMV (Fig. 5A) and cell-type specific KO lines (Fig. 5B & C). This point of inter-experimental variability should be mentioned and discussed briefly in the manuscript.

- Are efficiencies of primers used in the qRTPCR around 100% (80-120%)? That would be a mandatory requirement to use de ddCT method. Otherwise, the use of a standard curve is required.
